# University students’ resilience in post-pandemic period: a socio-ecological perspective

**DOI:** 10.3389/fpsyg.2025.1574153

**Published:** 2025-05-09

**Authors:** Jianjun Sheng, Davy Tsz Kit Ng, Peiyao Tian, Zhizi Zheng

**Affiliations:** ^1^Student Affairs Department, Zhejiang Agriculture and Forestry University, Hangzhou, China; ^2^Department of Mathematics and Information Technology, The Education University of Hong Kong, Tai Po, Hong Kong SAR, China; ^3^Faculty of Education, The University of Hong Kong, Pokfulam, Hong Kong SAR, China

**Keywords:** ecological system theory, influencing factor analysis, resilience, structural equation modeling (SEM), university students

## Abstract

**Introduction:**

The psychological impact of the COVID-19 pandemic on university students has become a significant concern, leading to increased attention on students’ mental health. In China, universities have recognized the importance of this issue and investigated students’ resilience in the post-pandemic world.

**Methods:**

This study utilized a quantitate research method to examine university students’ resilience and the factors influencing it. Employed purposive sampling, 1735 students from 5 universities in China participated in the research. A comprehensive questionnaire was distributed to collect data on participants’ demographic information, socio-ecological factors, and resilience levels.

**Results:**

Using the data analysis approach of descriptive statistics, independent sample *t*-test and structural equation modeling, the results revealed the following findings: (1) The majority of students demonstrated a moderate level of resilience (*M* = 2.949 out of 5, *SD* = 0.569). (2) Significant differences in resilience levels were observed among students based on demographic factors of gender and students’ leadership experience. (3) Regarding ecological factors, individuals were identified as the most influential factor on resilience levels, followed by family, school, and social factors. Among Individual factors, emotional regulation and coping abilities are the greatest influence.

**Discussion:**

Based on the results, the study provides targeted recommendations and strategies and addresses the identified factors to enhance students’ psychological resilience in university settings in the post-pandemic era.

## 1 Introduction

In the current social context, university students face unique and severe psychological challenges. Research shows that the levels of depression, anxiety, and stress reported by university students are significantly higher than those of the general population (Morrison and Pidgeon, 2017; Aylie et al., 2020). Multiple academic burdens, the pressure of adapting to new environments, and anxiety about uncertain futures all have profound impacts on the mental health of university students (Brand and Schoonheim-Klein, 2009; Galante et al., 2018). These difficulties have been exacerbated in the context of the COVID-19 pandemic (de Miranda et al., 2020). Giusti et al. (2020) found that Italy university students considered pandemic as a traumatic psychological distress. Giusti et al. (2021) further explored and confirmed the influence of distance education on university students’ mental health, social cognition, and memory abilities during the pandemic, which can also influence their academic performance. Celia et al. (2024) also found that the pandemic harmed university students’ psychological wellbeing. The pandemic forced universities to implement remote teaching, requiring many students to adapt to a new mode of learning from home (Biwer et al., 2021). This undoubtedly increased the learning difficulties and burdens for students who need to focus on their studies. Additionally, isolation and social distancing measures deprived students of face-to-face interactions with peers and instructors, reducing the social and emotional support available in campus life (Manchia et al., 2022). Furthermore, the uncertainty regarding job prospects due to the pandemic has heightened anxiety and stress levels, particularly among upperclassmen (Killgore et al., 2020). The lack of normal social interactions, combined with concerns about the future, has led to the emergence of mental health issues such as depression and anxiety among some university students. Current studies indicate that students who have experienced the pandemic exhibit higher levels of negative emotions (such as fear, depression, stress, and anxiety) and heightened risk perception (Alsolais et al., 2021; Hunt et al., 2021).

In the methods for alleviating negative psychological emotions among students, resilience plays a crucial role. Resilience is commonly defined as an individual’s capacity to maintain a positive emotional state, effectively navigate challenges, and ultimately recover and grow from adversity or stress (American Psychological Association, 2019). Resilience enables individuals to better manage their emotions, reducing the incidence of psychological problems such as anxiety and depression, and is a key factor in personal growth (Wu et al., 2020). Research indicates that university students’ resilience is closely linked to their mental health status (Abulfaraj et al., 2024; Dalmış et al., 2025; Fan and Liu, 2024). Enhancing resilience can help students cope more effectively with stress and challenges, thereby lowering the risk of anxiety and depression. This capacity allows students to maintain a positive mindset when facing academic and life difficulties, proactively seeking solutions instead of being trapped in negative emotions. Therefore, cultivating and enhancing university students’ resilience should be a primary goal of mental health intervention measures (Li and Hasson, 2020).

Some researchers have explored the status of students’ resilience levels (Pidgeon et al., 2014), methods for improving students’ resilience (Ungar et al., 2014), and factors that may affect students’ resilience (Erdogan et al., 2015). There are also studies that have used qualitative interviews (Ang et al., 2022) and quantitative analysis (Quintiliani et al., 2022) to explore students’ resilience during the pandemic. However, as the post-pandemic era progresses, the world has recovered and returned to normal, and many industries have also undergone changes after the pandemic, with the emergence of artificial intelligence and automation technologies (Agarwal et al., 2022). Digital transformation and technological capabilities have become more important than ever before (Lu et al., 2022). Recent reviews have pointed out that in the post-pandemic era, unemployment, family dynamics, socioeconomic status, social reorganization, and industrial change have been perceived among learners (Klimczuk et al., 2022). Therefore, it is necessary to explore the resilience levels of university students in the post-pandemic era, as well as the factors influencing their resilience levels.

Ecological systems theory posits that human society is built upon complex interdependent relationships among individuals, and environmental factors such as families, schools, and society (Stanley and Kuo, 2022). At the developmental level, this theory highlights that students’ growth is influenced by their surrounding social environments, including family settings, school culture, and social systems (Jiang et al., 2024). Therefore, to understand and foster students’ resilience, it is essential to consider individual traits as well as their interactions with family environments, school cultures, and societal structures (Zhang et al., 2022). Based on this theory, this study adopts a socio-ecological perspective to examine the levels of student resilience in the post-pandemic era, focusing on demographic differences, and the impact of individual factors (such as knowledge, skills, and values), family support, school belonging, and social factors on student resilience. Also, this study hypothesizes that individual traits mediate the relationship between family school and society factors and student resilience. By exploring these relationships, we aim to uncover how enhancing individual traits can strengthen students’ resilience, thereby providing valuable insights for educational practices. Specifically, the hypotheses of this study are:

H1 students’ resilience level shows difference among demographic factors.

H2 students’ resilience is predicted by school, society, family and society factors.

H3 Individual factors have mediate effect among the influence of school, family and society factors on students’ resilience levels.

## 2 Literature review

### 2.1 Definitions and types of resilience

Resilience is a crucial factor associated with the adaptation of students to their university environments (Pidgeon et al., 2014). Studies have demonstrated that higher levels of resilience are linked to a reduced risk of psychological distress, better management of academic demands, improved academic outcomes, and effective coping strategies when faced with academic pressures (Abbott et al., 2009). Lower levels of resilience can make university students vulnerable to negative impacts, leading to mental health, increased psychological distress, and greater adjustment difficulties (Varma et al., 2021). Previous research on resilience has predominantly focused on individuals experiencing short and long-term adversities. For example, university students may face academic failure, relationship affairs, and environmental pressures (Lee, 2017).

While there is no universal definition of resilience, it is widely recognized as an individual’s capacity to overcome adversities and successfully adapt to their environment (Windle, 2011). According to a review by Vella and Pai (2019), resilience is frequently described as the ability to recover and regain stability after facing challenges. The link between stress, negative life events, and the development of mental illness has long been acknowledged. These positive responses or outcomes in the face of significant risk or adversity are commonly referred to as resilience. Resilience is a construct within positive psychology that has been extensively studied for many years, predating the specific needs arising from the pandemic. It focuses on identifying personal qualities that empower individuals to thrive and flourish when faced with adversity. Other studies have similar definitions and refer to it as the ability to respond effectively and achieve success in the face of challenges during difficult times (e.g., Isaacs, 2014). Moreover, resilience has been recognized as a protective buffer that shields individuals from the negative impact of adversity (Mosanya, 2021). To this end, prior research indicates that higher levels of resilience in the university environment are associated with improved mental health, as well as successful transition and adjustment to university life. Therefore, it is important to study students’ resilience levels, investigate the elements causing this issue, and propose strategies to alleviate their negative psychological effects.

### 2.2 Pandemic research about students’ resilience

During the pandemic, there has been a heightened focus on students’ resilience, as the challenges and disruptions brought about by the global health crisis have had a significant impact on their well-being. Recent studies have aimed to investigate students’ psychological distress, resilience, and perceived social support across different countries. These studies provide valuable insights into the experiences of students during this challenging time and shed light on the factors that contribute to their resilience and overall mental well-being. For example, Quintiliani et al. (2022) conducted a study involving 955 students and revealed that 89.4% of participants experienced increased perceived stress, with 66% reporting moderate stress and 23.4% reporting high stress levels. More than half of the students reported decreased attention span and difficulties in studying, which raised concerns about their exam outcomes. The study underscored the positive impact of resilience skills in managing stressful events, particularly the challenges posed by the pandemic on students’ academic studies and interpersonal relationships. In a qualitative study by Ang et al. (2022), the resilience of undergraduate students in Singapore during the COVID-19 pandemic was examined. Intrinsic factors such as the desire to succeed and motivation were found to be crucial in fostering resilience. Extrinsic factors, including support from friends, family, teachers, and religion, were also identified as significant sources of resilience for students during the challenging times of the pandemic.

In China, in line with the growing recognition of the importance of university students’ mental health, the government has intensified its attention to this issue. Notably, the Ministry of Education issued a policy report titled “Comprehensive Strengthening and Improvement Plan for Student Mental Health Work in the New Era (2023–2025).” Higher education institutions need to pay attention to students’ mental health and psychological hygiene in the post-pandemic era. In China, studies have been conducted to explore students’ psychological conditions and the factors influencing anxiety among university students. Results showed that their anxiety related to the pandemic could be explained by the effect of the pandemic on their studies and concern for future employment in the post-pandemic world (Cao et al., 2020). Although our world has been gradually returning to a sense of normalcy, the post-pandemic period has been characterized by significant changes in various dimensions, including the emergence of industrial downturn, the rise of automated technologies, and the acceleration of digital transformation (Agarwal et al., 2022; Lu et al., 2022; Klimczuk et al., 2022). These changes have had a profound impact on different sectors, creating new challenges and uncertainties for students in their studies and future careers. Consequently, it becomes essential to investigate the resilience of students and analyze how ecological factors and support systems influence their perceptions and experiences in navigating these evolving circumstances.

### 2.3 Ecological system theory

Recent studies have used Bronfenbrenner’s ecological systems theory (EST) to conduct public mental health and psychological studies (Lane et al., 2023; Ryan and Barber, 2022). According to EST, it is important to examine and understand beliefs and behaviors by considering the multiple contexts in which individuals are situated. This perspective emphasizes that these beliefs and behaviors are not solely determined by an individual’s personal attributes. Instead, individuals exist within a series of interacting environmental systems, which can be categorized as microsystems, mesosystems, exosystems, and macrosystems. The issue of resilience can be discussed at the individual and societal level.

Previous research concerning the mental health of children and adolescents indicates that the EST frequently influences their development (Li et al., 2022; Liu et al., 2021). For example, Liu et al. (2021) suggested conducting multilevel modeling studies to the current studies to examine an ecological model of school engagement among middle school students, involving a sample of 19,084 participants across provinces in China. Multilevel modeling is employed to predict adolescents’ school engagement, considering both individual-level factors such as gender and family socioeconomic status (SES), as well as provincial-level factors, including economy, public cultural facilities, technological industry, and education. During the pandemic, some studies have identified the impact of combined interpersonal, peer, and cultural factors on psychosocial distress, bullying victimization, and worries (Zhang and Jia, 2023). In addition, other factors like poor capabilities to deal with stressful situations, pressures arising from career development, and job hunting (Ye et al., 2022).

This study aims to explore the impact of these contextual and social factors on various dimensions, including behavior, emotion, and cognition; however, many researchers focused on empowering other disenfranchised communities (Kim et al., 2019) and suggested the use of photovoice with underrepresented and understudied populations. Some researchers also used photovoice with Muslim participants to explore their experiences related to different specific topics including physical exercise and women’s role (Bromfield and Capous-Desyllas, 2017; Chakraborty, 2009; Eyres et al., 2019; Miled, 2019; Murray et al., 2015; Reimers, 2016; Samsuni et al., 2019) and high school students in education (Roxas and Vélez, 2019). Similarly, other researchers reported that photovoice enables the acquisition of people’s experiences more accurately as they can identify, represent, and enhance their own or their community’s status through captions, explanations, and photos (Haugen et al., 2019; Nitzinger et al., 2019; Sullivan, 2017).

### 2.4 Theoretical foundations

According to the Ecological Systems Theory, individuals have an innate ability to interact with the environment, and there is a mutual benefit and harmony between people and their surroundings. Individual behavior is purposeful, and their environment shapes the meaning of individuals (Lane et al., 2023; Ryan and Barber, 2022). Therefore, understanding individuals requires considering their environmental context. Individual problems are rooted in life experiences and understanding and judgment should be made within this context. This study explores the key ideas of the EST, which emphasizes the interconnectedness of various environmental systems. These systems include microsystems (i.e., individual knowledge, skills, and attitudes) and macrosystems (i.e., family influence, school influence, and societal adaptability).

The microsystem influences encompass individual factors such as knowledge, skills, and attitudes (Schraagen et al., 2011). In terms of knowledge, students can access relevant resources, courses, and training that enhance their resilience. This knowledge equips them with the ability to utilize their own experiences as well as draw from the experiences of others to effectively navigate through difficulties (Helling and Chandler, 2021). As students encounter setbacks and challenges, they embrace them as opportunities for personal growth, leading to increased maturity and ability to cope with adversity (Wong et al., 2022). In terms of skills, students demonstrate the capacity to regulate their emotions efficiently (McCloughen and Foster, 2018) and adapt to different situations (Mayordomo et al., 2021). Clear goals guide their lives, and they maintain unwavering determination to pursue them, persisting even in the face of obstacles. Students have honed their problem-solving skills, formulating and executing comprehensive plans step by step. Regarding values, students view challenges and setbacks as integral components of life experiences, recognizing that adversity is a powerful motivator, propelling individuals to strive for excellence and reach their fullest potential (Walsh, 2015).

Students’ family environment, including family expectations, support, and relationships, significantly impacts their resilience and ability to cope with adversity (Wong et al., 2022). Disharmonious family relationships or parenting styles can increase psychological burden and stress, potentially leading to psychological crises (Tavassolie et al., 2016). Students’ development and relationships can be shaped by family expectations, fostering their autonomy and positive parent-adolescent relationships (Bi et al., 2018). Family support also plays a crucial role in meeting students’ needs and providing emotional and psychological backing. Parents’ communication skills and minimal conflicts create a harmonious family environment (Guiffrida and Douthit, 2010).

School is a critical contextual environment for college students outside of their families. Students must find the right self-development path, adopt effective learning methods, and maintain a regular routine. Lack of planning and long-term goal setting can lead to crises in academic performance, emotions, social relationships, and overall life development (Gueldner et al., 2020). Peer relationships also play a role in college students’ physical and mental development. Particularly for freshmen, investing time and energy in social relationships is common to overcome feelings of unfamiliarity and adapt to their new roles (Uslu and Gizir, 2017). However, this can also increase pressure and frustration when facing setbacks. To sum up, a healthy campus environment, a supportive classroom atmosphere, and equal development opportunities significantly influence students’ psychological well-being (Zee and Koomen, 2016).

**Figure 1:**
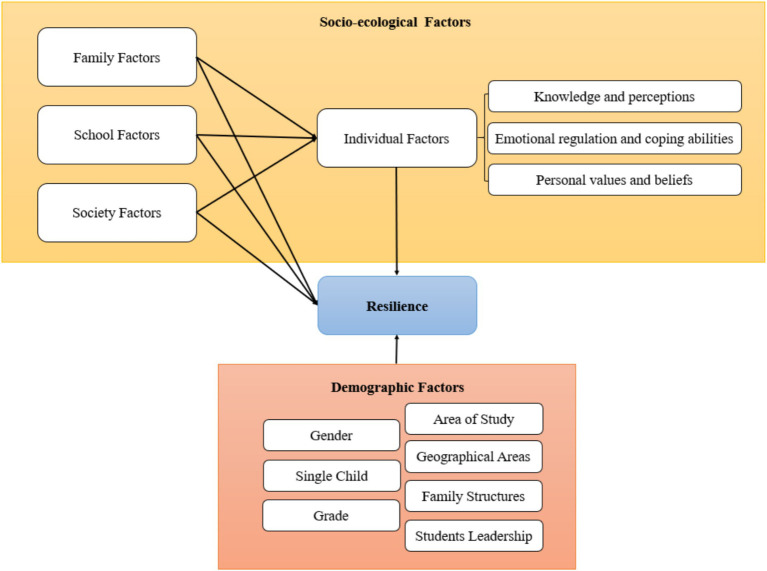
Theoretical framework of this study.

Social factors play a significant role in students’ lives, providing them with the necessary support and resources (Navarro and Tudge, 2023). Firstly, students can turn to others for help through online platforms, seeking assistance and guidance when facing difficulties. At a macro level, students can feel the social support provided by important individuals in their lives, such as counselors, doctors, and volunteers. They often engage in social group activities, fostering a sense of community and connection. Additionally, students benefit from strong relationships with their relatives, experiencing understanding, support, and care from one or more family members. They trust and rely on their relatives for guidance and assistance. Peer support is also valuable, as students have peers with whom they can share their difficulties and seek advice. They engage in discussions with classmates and friends to find solutions to problems. Moreover, students draw inspiration and strength from unfamiliar peers who serve as role models. These social factors, including seeking help from others, social support from important individuals, active participation in social activities, strong relationships with relatives, and peer support, contribute to students’ well-being, resilience, and personal growth. Figure 1 summarizes the sub-components of socio-ecological domains, displays the theoretical framework that interplays between the resilience of college students and their ecological systems, and serves as the foundation for questionnaire development.

This study explores the relationship between the resilience of college students, highlighting the significance of understanding individuals and considering the influence of various ecological factors on their well-being. Based on the EST, Figure 1 shows that the proposed model of this study examines the interactions between the demographic, individual, external environment factors (family, school, and society), and resilience level. These predictors may affect student resilience at the high school level. They are the combined results of previous studies derived from the EST.

### 2.5 Theoretical foundations

Most of the articles in this field have primarily focused on studying the immediate or ongoing impacts of COVID-19. However, only a few studies have delved into the long-lasting psychological effects of the pandemic and examined students’ well-being months after the initial outbreak (e.g., Wu et al., 2020). Additionally, other countries such as Italy, Spain, and Singapore have conducted similar research on resilience and psychological impacts to explore the psychological influences of the pandemic (Quintiliani et al., 2022). Therefore, it is necessary to update the situation further and explore the resilience and psychological impacts specific to the Chinese context, especially in the post-pandemic era. To address these research gaps, the present study aimed to investigate the levels of resilience among students in Zhejiang Province, specifically after China officially ended its lockdown policy in January 2023. The study examined how students’ resilience levels were impacted and potentially changed as they transitioned from the pandemic period to the post-pandemic phase. First, factors influencing resilience and potential differences in resilience levels based on gender, year of study, and the status of being a single child were explored. Also, this study aimed to identify the factors contributing to psychological resilience among university students. Based on the research objectives, two research questions were identified:

RQ1a. What is the overall extent of resilience among university students?

RQ1b. How does university students’ resilience vary across different demographic groups?

RQ2. What underlying socio-ecological factors (i.e., individual, family, school, and society) contribute to students’ resilience? What is the underlying influencing mechanism?

## 3 Methodology

### 3.1 Participants

This study distributed online questionnaires to students who voluntarily signed up to participate from 5 universities in Zhejiang Province, China. A total of 1735 questionnaires were collected, with a response rate of 95.9%. The electronic questionnaire was designed to permit submission only upon the completion of all items, thereby ensuring the integrity and completeness of the responses. The demographic characteristics of the sample are shown in Table 1. The average grade level of the participants was 1.44 (SD = 0.822).

**Table 1:** Demographic characteristics.

Variables		%	*N*
Gender	Male	40.7	707
	Female	59.3	1,028
Area of Study	Business and Economics	3.6	62
	Law	6.2	107
	Education	0.4	7
	Literature and Social Science	10.2	176
	Science	5.0	87
	Engineering	29.9	518
	Agriculture	15.0	261
	Medicine	0.3	6
	Management	16.3	282
	Arts	13.2	229
Single child	Yes	39.4	684
	No	60.6	1,051
Year of Study	Year 1	48.8	847
	Year 2	23.9	415
	Year 3	21.0	365
	Year 4 or above	6.2	108
Geographical areas	Rural areas	46.2	801
	Urban areas	53.8	934
Family structures	Nuclear family	1,378	1,369
	One-parent family	115	95
	Remarried family	66	82
	Extended family	176	189
Student leadership	Currently work in a student association	37.8	655
	No experience	25.2	438
	Had worked in a student association	37.0	642

During the questionnaire distribution, the research team ensured the voluntariness of the participants and did not exert any compulsion or undue influence on any student.

### 3.2 Instruments design and development

#### 3.2.1 Questionnaire design

This study developed a Resilience and Ecological Questionnaire (REQ) to measure students’ resilience and socio-ecological factors. The questionnaire was guided by the resilience measurement approach developed by Turner et al. (2017) in the university context. We aimed to investigate patterns of student resilience and analyze the influencing factors of student resilience from a socio-ecological perspective. According to social-ecological theory, the development of individual students is a complex system that includes individual, family, school, and societal factors (Coulombe et al., 2020). Therefore, researchers made appropriate revisions to the questionnaire based on previous relevant research and added relevant descriptive statements related to socio-ecological factors (Connell et al., 2010). Additionally, this study also included seven demographic-related items in the questionnaire to comprehensively explore the impact of demographic factors on student resilience.

The final questionnaire developed in this study consists of 6 sub-questionnaires, where the demographic-related items (7 items) are presented in a single-choice format, and the remaining items on resilience level (14 items), individual factors (9 items), family factors (9 items), school factors (10 items), and society factors (8 items) are presented in a 5-point Likert scale format, with a total of 57 items.

#### 3.2.2 Reliability test

To ensure the quality of the questionnaire, a reliability analysis was conducted on the items in the questionnaires. The Cronbach’s alpha coefficients of the sub-questionnaire of Resilience level (0.961), Individual factor (0.938), Family factor (0.964), School factor (0.957), and Society factor (0.942) were greater than 0.80. This indicates that the questionnaire demonstrates internal consistency.

#### 3.2.3 Confirmatory factor analysis

To verify the validity of the questionnaire, the study conducted a confirmatory factor analysis.

##### 3.2.3.1 Model fit

First, the overall data was subjected to the Kaiser-Meyer-Olkin (KMO) and Bartlett’s sphericity tests. The results showed that the KMO value was 0.981, and Bartlett’s sphericity test value was 98624.02 with a *p*-value of 0.000 (less than 0.05), indicating a high level of sphericity and the rejection of the null hypothesis. This suggests that the variables in the analysis have correlations, indicating the appropriateness of factor analysis. Based on this, the model fit analysis of the questionnaire was conducted. CMIN/DF = 9.492 (<10), the values of CFI (0.894), TLI (0.889), and IFI (0.894), are all greater than 0.89, and RMSEA (0.07) and RMR (0.018) are both less than 0.08 (Kim, 2005). Therefore, in this study, the model fit of the questionnaire is good.

##### 3.2.3.2 Convergent validity and composite reliability

Building upon the good model fit, the questionnaire is further analyzed for convergent validity and composite reliability (CR) (Table 2). Convergent validity refers to the consistency among multiple items measuring the same concept, and it is assessed through the Average Variance Extracted (AVE) statistic. To achieve good convergent validity, the AVE value should be 0.50 or higher, indicating that the latent variable accounts for at least 50% of the variance in its indicators. Composite reliability is a measure of the internal consistency of the construct, and the higher the value, the more reliable the construct. Generally, CR values above 0.70 indicate good reliability of the construct. As shown in Table 2, the CR values of each sub-questionnaire in this study are greater than 0.7, and the AVE values are greater than 0.5, indicating that the questionnaire has good convergent validity and composite reliability.

**Table 2:** Factor loading, CR, and AVE of the questionnaire.

Path			Estimate	CR	AVE
F1	<−--	Family factor	0.865	0.96	0.75
F2	<−--	Family factor	0.897		
F3	<−--	Family factor	0.826		
FF4	<−--	Family factor	0.892		
FF5	<−--	Family factor	0.839		
FF6	<−--	Family factor	0.897		
FF7	<−--	Family factor	0.896		
FF8	<−--	Family factor	0.828		
FF9	<−--	Family factor	0.856		
SF1	<−--	School factor	0.715	0.96	0.72
SF2	<−--	School factor	0.794		
SF3	<−--	School factor	0.819		
SF4	<−--	School factor	0.845		
SF5	<−--	School factor	0.860		
SF6	<−--	School factor	0.895		
SF7	<−--	School factor	0.907		
SF8	<−--	School factor	0.889		
SF9	<−--	School factor	0.886		
SF10	<−--	School factor	0.856		
SOF1	<−--	Society factor	0.746	0.94	0.67
SOF2	<−--	Society factor	0.826		
SOF3	<−--	Society factor	0.787		
SOF4	<−--	Society factor	0.831		
SOF5	<−--	Society factor	0.841		
SOF6	<−--	Society factor	0.834		
SOF7	<−--	Society factor	0.856		
SOF8	<−--	Society factor	0.833		
IF1	<−--	Individual factor	0.613	0.94	0.64
IF2	<−--	Individual factor	0.852		
IF3	<−--	Individual factor	0.861		
IF4	<−--	Individual factor	0.805		
IF5	<−--	Individual factor	0.838		
I6	<−--	Individual factor	0.821		
I7	<−--	Individual factor	0.794		
I8	<−--	Individual factor	0.835		
I9	<−--	Individual factor	0.802		
R1	<−--	Resilience level	0.791	0.96	0.64
R2	<−--	Resilience level	0.827		
R3	<−--	Resilience level	0.834		
R4	<−--	Resilience level	0.815		
R5	<−--	Resilience level	0.783		
R6	<−--	Resilience level	0.863		
R7	<−--	Resilience level	0.809		
R8	<−--	Resilience level	0.730		
R9	<−--	Resilience level	0.852		
R10	<−--	Resilience level	0.800		
R11	<−--	Resilience level	0.797		
R12	<−--	Resilience level	0.744		
R13	<−--	Resilience level	0.803		
R14	<−--	Resilience level	0.751		

##### 3.2.3.3 Discriminant validity

This study used the Fornell-Larcker criterion to measure the discriminant validity of the questionnaire. This criterion requires that the AVE value of each latent variable should be greater than the square of the correlation coefficient between that latent variable and other latent variables (Table 3). If this condition is met, the scale has good discriminant validity. From Table 3, the correlation coefficients of the various sub-questionnaires are all less than the square root of the AVE value, indicating that the overall discriminate validity of this questionnaire is good.

**Table 3:** Discriminant validity.

Variable	Resilience level	Individual	Family	School	Society
Resilience level	***0.800***				
Individual	0.796^***^	***0.800***			
Family	0.623^***^	0.649^***^	***0.866***		
School	0.636^***^	0.661^***^	0.633^***^	***0.849***	
Society	0.631^***^	0.662^***^	0.657^***^	0.698^***^	***0.819***

Italicized and bold text represents the square root of AVE.

***p < 0.001.

### 3.3 Data analysis

After the Data Collection, The Reliability test in SPSS 27.0 and the Confirmatory factor analysis in Amos 27.0 were conducted to ensure the quality of the questionnaire and the data. Based on the good quality of the data, to address RQ1a, a descriptive analysis was conducted to examine students’ overall resilience levels. Also, to identify the demographic differences among students’ resilience, this study conducted a normal distribution test first. If the data is normally distributed, independent t-test and ANOVA can be used and if the data are not normally distributed, the non-parametric tests can be considered as an alternative method. In our study, the data followed the normal distribution (using skewness and kurtosis). Therefore, independent t-tests and ANOVA were employed to understand which demographic groups tend to have higher perceptions of resilience (RQ1b). For RQ3, Structural Equation Modeling were conducted to explore how well the individual, family, school, and society factors predicted students’ resilience levels and the influencing mechanism.

## 4 Results

### 4.1 Students’ overall resilience level

The overall level of students’ resilience was calculated, first. Descriptive statistics showed that students mean resilience is 2.949 (SD = 0.569) out of 5. It reveals that most students have a moderate level of resilience. In addition, the skewness and kurtosis values show that the students’ overall resilience data is normally distributed.

### 4.2 Students’ resilience difference among different demographic groups

The study further explored the effects of 7 demographic factors—gender, area of study, single-child situation, year of study, geographical areas, family structures, and student leadership—on the students’ resilience and socio-ecological factors by conducting independent sample tests and ANOVAs.

Independent sample *t*-tests informed the effects of Gender, area of study, and single-child situation on their resilience levels. For gender, there was a significant difference in the overall resilience scores for male (*M* = 2.99, SD = 0.65) and female students (*M* = 2.92; SD = 0.50); *t*(1257.287) = 2.587, *p* = 0.01 (<0.05). However, for the geographical areas of their families, there was no significant difference in the overall resilience scores for students in rural areas (*M* = 2.92, SD = 0.54) and urban areas (*M* = 2.98; SD = 0.60); *t*(1622.017) = 1.947, *p* = 0.052 (>0.05). Also, for single child situation, there was no significant difference in the overall resilience scores for students who are single child (*M* = 2.98, SD = 0.57) and no single child (*M* = 2.93; SD = 0.57); *t*(1733.000) = 1.757, *p* = 0.079 (>0.05).

Also, ANOVAs informed the differences in overall resilience did not reach statistical significance (*p* > 0.05) in study area (*F* = 1.611, *p* = 0.107), grade (*F* = 0.251, *p* = 0.861), and family structure (*F* = 1.595, *p* = 0.189). On the other hand, in the student’s leadership, the difference in overall resilience was significant (*F* = 13.173 *p* = 0.000 < 0.001). Based on the ANOVA results, we further compared the differences in resilience among students with different student leadership experiences. The mean difference between “Had worked in a student association” and “Currently work in a student association” is 0.05 (*p* = 0.193). The mean difference between “Had worked in a student association” and “No experience” is 0.16 (*p* < 0.001). The mean difference between “Currently working in a student association” and “No experience” is 0.11 (*p* = 0.001). In other words, students with no prior student association work experience have significantly lower resilience levels than those who have worked or are currently working in a student association. However, there is no significant difference in resilience factors between students who have previously worked in a student association and those who are currently working in one.

### 4.3 Socio-ecological factors that contribute to resilience

#### 4.3.1 Predictive power of socio-ecological factors on resilience

**Figure 2:**
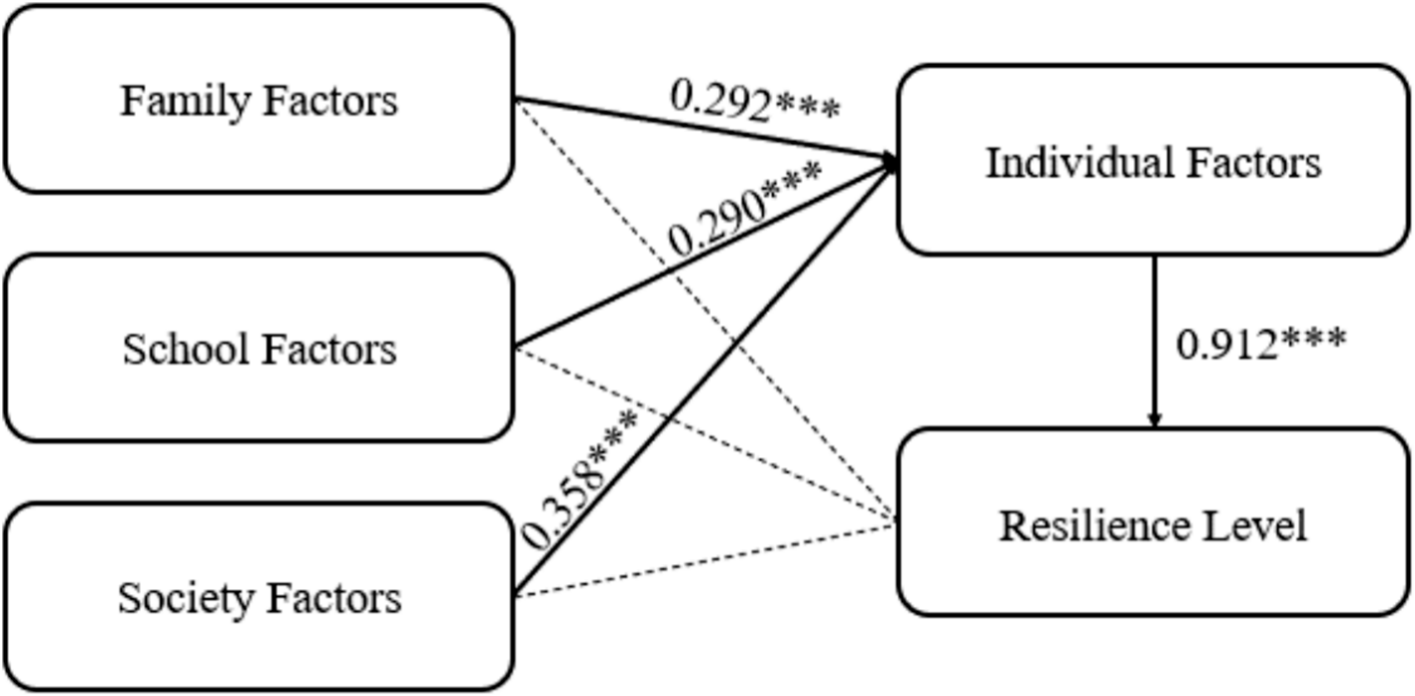
Path analysis results. ***p < 0.001.

To explore the influence of individual, family, and social factors on students’ resilience levels, the study conducted a path analysis. As shown in Figure 2 and Table 4, individual factors have a significant direct positive impact on students’ resilience, while the direct effects of family, school, and societal factors on resilience are not significant. However, these three external environmental factors all indirectly influence students’ resilience levels through their impact on individual factors. This indicates that the effects of external environmental factors on resilience are primarily realized by influencing students’ own cognition, emotions, and abilities.

**Table 4:** Path analysis of first and second order model.

First order model path			Estimate	S.E.	C.R.	*p*
Individual factor	<−--	Family factor	**0.292**	0.022	12.240	***
Individual factor	<−--	School factor	**0.290**	0.034	9.133	***
Individual factor	<−--	Society factor	**0.358**	0.036	10.432	***
Resilience level	<−--	Individual factor	**0.912**	0.035	27.063	***
Resilience level	<−--	Family factor	0.004	0.020	0.172	0.863
Resilience level	<−--	School factor	0.010	0.029	0.363	0.717
Resilience level	<−--	Society factor	0.017	0.032	0.597	0.551

Second order model path			Estimate	S.E.	C.R.	*p*
Knowledge and perceptions	<−--	Family factor	0.266***	0.025	9.084	***
Emotional regulation and coping abilities	<−--	Family factor	0.278***	0.026	9.988	***
Personal values and beliefs	<−--	Family factor	0.240***	0.025	9.080	***
Knowledge and perceptions	<−--	School factor	0.155***	0.037	4.105	***
Emotional regulation and coping abilities	<−--	School factor	0.207	0.040	5.605	***
Personal values and beliefs	<−--	School factor	0.284	0.039	7.926	***
Personal values and beliefs	<−--	Society factor	0.398	0.041	10.211	***
Emotional regulation and coping abilities	<−--	Society factor	0.428	0.044	10.426	***
Knowledge and perceptions	<−--	Society factor	0.477	0.043	10.709	***
Resilience level	<−--	Family factor	−0.022	0.022	−0.960	0.337
Resilience level	<−--	School factor	0.025	0.030	0.889	0.374
Resilience level	<−--	Society factor	−0.052	0.039	−1.415	0.157
Resilience level	<−--	Knowledge and perceptions	0.545	0.037	16.134	***
Resilience level	<−--	Emotional regulation and coping abilities	0.360	0.028	12.768	***
Resilience level	<−--	Personal values and beliefs	0.168	0.026	6.335	***

***p < 0.001.

#### 4.3.2 Mediation roles of individual factors on resilience

**Figure 3:**
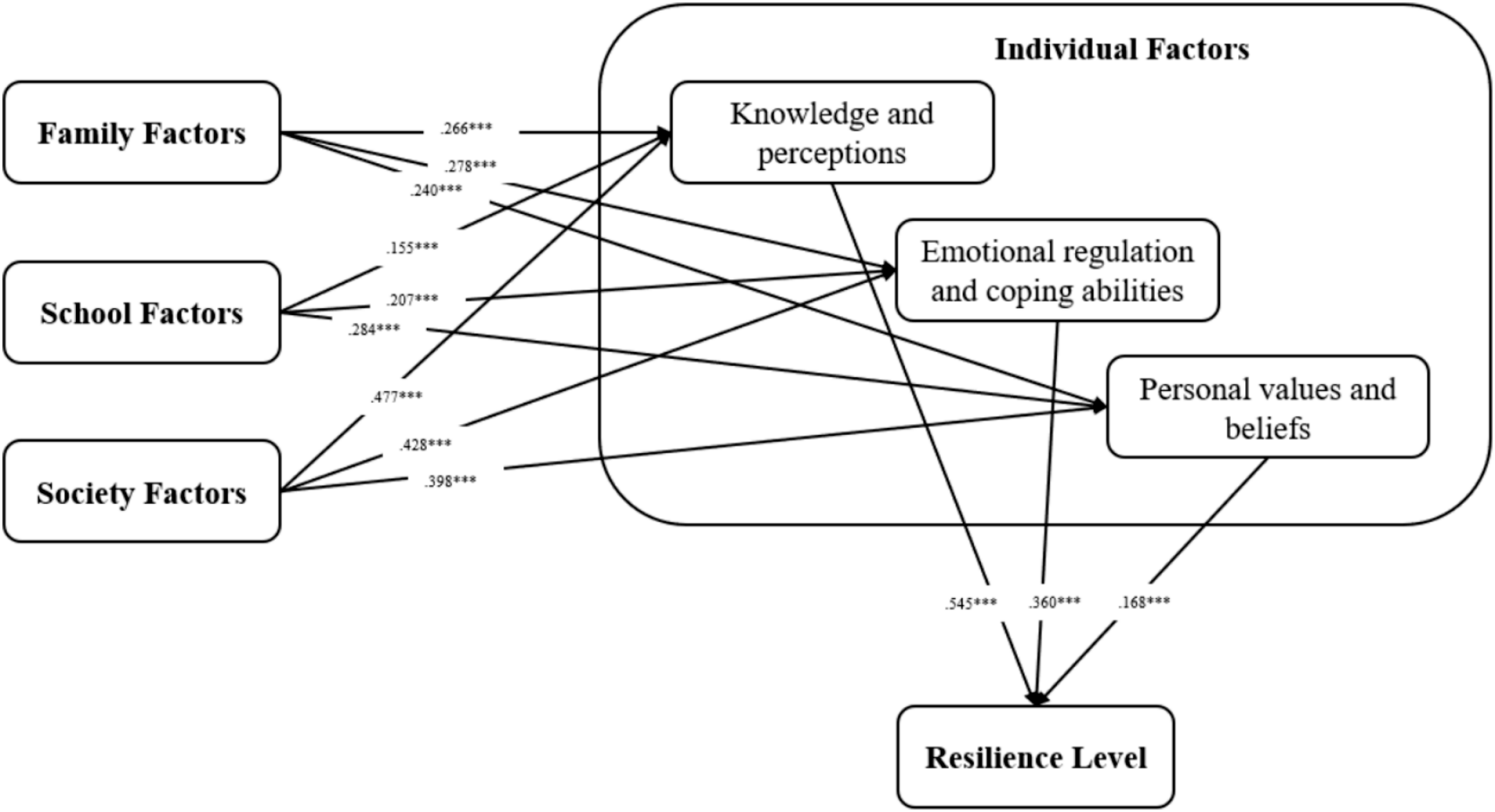
Second order model. ***p < 0.001.

Since individual factors have a huge impact on students’ resilience, and they almost completely mediate the influence of external environmental factors such as family, school, and society on resilience, this study further explored the mediation mechanism of the different categories of individual factors. In the individual factors’ sub-questionnaire, items 1, 2, and 3 examined cognition, items 4, 5, and 6 examined ability, and items 7–9 examined emotion and beliefs. The Second order model can be seen in Figure 3 (Second order model fit: CMIN/DF = 9.838, CFI = 0.891, TLI = 0.884, IFI = 0.891, RMR = 0.023, RMSEA = 0.071). As shown in Table 4, students’ knowledge and perceptions, emotional regulation and coping abilities, and personal values and beliefs all had a significant direct impact on their resilience. Among them, knowledge and perceptions had the greatest influence, followed by emotional regulation and coping abilities, and personal values and beliefs. Furthermore, knowledge and perceptions, emotional regulation and coping abilities, and personal values and beliefs all played a significant mediating role in the influence of social, family, and school factors on students’ resilience.

## 5 Discussion

There is existing literature that highlights the various psychological effects that public health emergencies can have on college students (Quintiliani et al., 2022). In the post-COVID-19 world, universities worldwide have taken measures such as offering more students’ psychological health services to alleviate their long-term psychological impacts brought by the pandemic, which may impact both the education and mental well-being of students (Gewin, 2020). This study aimed to assess the psychological status of university students during the post-pandemic world, and explore various socio-ecological factors that influenced their resilience levels, including gender, area of study, single child, year of study, family geographical areas and structures, and student leadership.

According to RQ1, the research results showed that after the pandemic and recovery measures, students experienced a moderate level of adaptability (2.949 out of 5 points). Subsequently, independent t-tests and ANOVA were used to examine the differences in adaptability among different demographic groups. The results revealed that female students exhibited lower resilience levels compared to their male counterparts. In contrast, Chen et al. (2021) found that emotional competence in preschool children significantly predicts their resilience, with gender showing little impact on emotional competence in that age group. This study’s findings are inconsistent with previous research, as significant gender differences were identified among the university student population. The age range of the sample may influence this outcome (Veijalainen et al., 2021). Empirical studies by Graves et al. (2021) indicate that female students in universities experience significantly higher levels of stress compared to their male peers. Additionally, research by Mezzalira et al. (2022) and Montolio and Taberner (2021) found that male university students perform better under high pressure than female students. Stress is an important factor affecting the dynamic process of resilience (Sun et al., 2022). Therefore, female students may have relatively lower resilience during their university years. Exploring the possible reasons for this discrepancy reveals that societal expectations often place more protective and dependent roles on women, while men are expected to be more independent (Nielson et al., 2020). These gender role differences may impact women’s opportunities and motivations to develop resilience. Furthermore, research indicates that women may be more susceptible to emotional influences, which could hinder their ability to maintain rationality and composure in challenging situations (García-Fernández et al., 2021). In addition to gender differences, the research also found that students’ experience as student leaders would also affect their level of resilience. Maykrantz and Houghton's (2020) research also found that self-leadership practices reduced students’ stress levels. Students who participated in more school leadership services tended to have higher levels of resilience. Serving as student leaders requires facing various challenges and difficulties, and in the process of solving problems and coping with stress, students can cultivate stronger resilience.

Regarding question 2, this study first analyzed the predictive power of four social-ecological factors (i.e., individual, family, school, and society) on students’ resilience using a first-order structural equation model. The results showed that individual factors had the greatest impact on students’ resilience, while the influence of family, society, and school was very small. Relevant literature research found that individuals undergo significant development in cognition, emotion, and identity during adolescence and youth. Therefore, compared to external environmental factors, individual factors may directly impact students’ resilience (Allen et al., 2023). The mediating effect results of this study showed that family, school, and society primarily influence students’ resilience by affecting their individual characteristics, further confirming and developing previous research findings. Furthermore, due to the significant mediating role of individual factors, and almost complete mediation, this study further explored the mediating effects of three different categories of individual factors: knowledge and perceptions, Emotional regulation and coping abilities, and Personal values and beliefs. The results showed that all three categories of individual factors significantly mediate the influence of family, society, and environment on resilience. Literature research found that a good family and school environment, among other external factors, helps students develop positive emotional regulation abilities, which may improve their ability to cope with difficulties (Eisenberg et al., 2010). Moreover, students are easily influenced by the behavior of family members, teachers, and others in society during their growth, learning coping skills and strategies (Li et al., 2023). In addition, educational resources, support services, etc., provided by families, schools, and society can affect students’ confidence and beliefs in coping with difficulties (Rice et al., 2013). These environmental influences on individual factors may further affect students’ resilience levels.

The findings of this study provide valuable insights for various stakeholders, including university administrators, educators, and students themselves. Recognizing that demographic characteristics, such as family structure or living situations, are beyond our control, it becomes essential to focus on providing additional support to help students manage academic-related stress and concerns about their future careers. One effective way to achieve this is by enhancing educational and psychological services. Counseling services, in particular, are crucial resources that provide psychological support during periods of academic difficulty (Celia et al., 2024). Research by Kivlighan et al. (2021) demonstrates that students who utilized counseling experienced improved GPAs, highlighting the positive impact of these services on academic success. To build on this, universities should ensure that counseling programs are accessible and well-structured, enabling students to manage stress and effectively navigate challenges (Giusti et al., 2020; Giusti et al., 2021). Furthermore, Universities can offer professional development opportunities for teachers to help them recognize and address student stress, as well as expand access to mental health services tailored to students’ needs. In addition, educators and mental health professionals, could collaborate to integrate resilience training into the curriculum, provide structured mentoring programs, and design targeted resilience-building workshops. Along the way, universities can take a proactive role in fostering academic success and holistic well-being of their students.

## 6 Conclusion and limitations

This study analyzes the resilience of university students in the post-pandemic era. The results find that their overall resilience is at a moderate level. Furthermore, there are significant differences in resilience based on gender and leadership experience. Specifically, male students exhibit significantly higher resilience than female students, and those currently or previously in student leadership roles demonstrate greater resilience compared to those without such experience. Using a socio-ecological perspective to examine the factors influencing student resilience, the research reveals that resilience levels are primarily predicted by individual factors. Society, school, and family factors mainly influence student resilience by impacting individual factors. A deeper analysis of the mechanisms influencing individual factors shows that knowledge and perceptions, emotional regulation and coping skills, as well as personal values and beliefs, all have a significant direct impact on resilience. Among these, knowledge and perceptions have the most substantial effect, followed by emotional regulation and coping skills, and finally personal values and beliefs. Additionally, knowledge and awareness, emotional regulation and coping skills, and personal values and beliefs play important mediating roles in the influence of social, family, and school factors on students’ psychological resilience.

While this study provides a general understanding of university students’ resilience in post-pandemic era, it has certain limitations. The data collected pertains to students from China, which needs future studies to examine whether this also applies in other geographical areas. Additionally, this study did not measure students’ resilience and psychological factors during or before the pandemic. It lacks accurate longitudinal analyses. This study only measures resilience across demographic groups and social-ecological factors could be made in the post-pandemic world. The study solely relied on online administration of questionnaires, and no in-person interviews were conducted. Future interviews are necessary to understand students’ feedback, understand their innate feelings, and triangulate the findings.

## Data Availability

The datasets presented in this article are not readily available because the data that support the findings of this study are available on request from the corresponding author. The data are not publicly available due to privacy or ethical restrictions. Requests to access the datasets should be directed to Peiyao Tian, ptian@connect.hku.hk.
