# Supplementary material for: University students’ resilience in post-pandemic period: a socio-ecological perspective
Source: Front Psychol. 2025 May 9;16:1574153. doi: 10.3389/fpsyg.2025.1574153 (PMC12098287; doi:10.3389/fpsyg.2025.1574153)
Supplement: Supplementary file 1 [file Supplementary_file_1.docx]

**Resilience and Ecological Questionnaire**

Dear Students,

We are conducting a study on enhancing the resilience of university students from an ecological systems perspective. This survey targets undergraduate students within Zhejiang Province and aims to collect relevant information to support research on improving students' resilience. There are no right or wrong answers to the questions in this questionnaire; please fill it out carefully based on your actual situation.

This questionnaire is anonymous, and we assure you that all information you provide will be kept strictly confidential and will not be disclosed to any individuals or organizations. Thank you very much for your support!

**Demographic Factor**

DF1. What is your gender? [Multiple choice]

1. Male
2. Female

DF2. What is your major? [Multiple choice]

1. Business and Economics
2. Law
3. Education
4. Literature and Social Science
5. Science
6. Engineering
7. Agriculture
8. Medicine
9. Management
10. Arts

DF3. What is your year of study? [Multiple choice]

1. Year 1
2. Year 2
3. Year 3
4. Year 4 or above

DF4. Where is your family located? [Multiple choice]

1. Rural areas
2. Urban areas

DF5. Are you an only child? [Multiple choice]

1. Yes
2. No

DF6. What is your family structure? Note: Nuclear family—consists of parents and children; Extended family—includes other relatives in addition to the nuclear family members. [Multiple choice] *

1. Nuclear family
2. One-parent family
3. Remarried family
4. Extended family

DF7. What is your current student work position? [Multiple choice]

1. Currently work in a student association
2. No experience
3. Had worked in a student association

**Resilience Level**

RL 1. I can respond to problems with a sense of humor.

A Strongly agree B Agree C Neutral D Agree E Strongly disagree

RL 2. I can handle difficulties when they arise.

A Strongly agree B Agree C Neutral D Agree E Strongly disagree

RL 3. I can adapt flexibly to changes.

A Strongly agree B Agree C Neutral D Agree E Strongly disagree

RL 4. Accumulating experiences makes me stronger.

A Strongly agree B Agree C Neutral D Agree E Strongly disagree

RL 5. After experiencing illness or hardship, I have strong resilience.

A Strongly agree B Agree C Neutral D Agree E Strongly disagree

RL 6. Even when facing obstacles, I can achieve my goals.

A Strongly agree B Agree C Neutral D Agree E Strongly disagree

RL 7. I can concentrate and think clearly under pressure.

A Strongly agree B Agree C Neutral D Agree E Strongly disagree

RL 8. I do not get discouraged by failure.

A Strongly agree B Agree C Neutral D Agree E Strongly disagree

RL 9. I consider myself a strong person when facing life's challenges.

A Strongly agree B Agree C Neutral D Agree E Strongly disagree

RL 10. I can manage unpleasant feelings, such as anger.

A Strongly agree B Agree C Neutral D Agree E Strongly disagree

RL 11. I can communicate and connect easily with others.

A Strongly agree B Agree C Neutral D Agree E Strongly disagree

RL 12. I can control my actions and avoid extremes regardless of my emotions.

A Strongly agree B Agree C Neutral D Agree E Strongly disagree

RL 13. I can always contribute to teams or groups.

A Strongly agree B Agree C Neutral D Agree E Strongly disagree

RL 14. I can usually identify others' emotions and needs.

A Strongly agree B Agree C Neutral D Agree E Strongly disagree

**Individual Factor**

IF 1. I have taken courses or training related to resilience enhancement.

A Strongly agree B Agree C Neutral D Agree E Strongly disagree

IF 2. I can use my own or others' experiences to cope with difficulties.

A Strongly agree B Agree C Neutral D Agree E Strongly disagree

IF 3. I generally become more mature and experienced after setbacks.

A Strongly agree B Agree C Neutral D Agree E Strongly disagree

IF 4. I can effectively adjust my emotions in a short time.

A Strongly agree B Agree C Neutral D Agree E Strongly disagree

IF 5. I have clear goals in life and can stick to them.

A Strongly agree B Agree C Neutral D Agree E Strongly disagree

IF 6. I can develop a plan to solve problems and execute it step by step.

A Strongly agree B Agree C Neutral D Agree E Strongly disagree

IF 7. I am willing to improve myself for the prosperity of my country and nation.

A Strongly agree B Agree C Neutral D Agree E Strongly disagree

IF 8. I view difficulties and setbacks as part of life experiences.

A Strongly agree B Agree C Neutral D Agree E Strongly disagree

IF 9. I believe adversity has a motivating effect on people.

A Strongly agree B Agree C Neutral D Agree E Strongly disagree

**Family Factor**

FF 1. My parents respect my opinions and allow me to develop naturally.

A Strongly agree B Agree C Neutral D Agree E Strongly disagree

FF 2. I feel that my parents try to make my youth meaningful and colorful.

A Strongly agree B Agree C Neutral D Agree E Strongly disagree

FF 3. I am willing to meet my parents' expectations and have made some progress.

A Strongly agree B Agree C Neutral D Agree E Strongly disagree

FF 4. My parents respect my thoughts and always encourage and care for me.

A Strongly agree B Agree C Neutral D Agree E Strongly disagree

FF 5. My parents often meet my material needs.

A Strongly agree B Agree C Neutral D Agree E Strongly disagree

FF 6. My parents have confidence in me and provide spiritual support.

A Strongly agree B Agree C Neutral D Agree E Strongly disagree

FF 7. I feel a warm, caring, and affectionate relationship with my parents.

A Strongly agree B Agree C Neutral D Agree E Strongly disagree

FF 8. My parents communicate well to solve problems and rarely argue.

A Strongly agree B Agree C Neutral D Agree E Strongly disagree

FF 9. Family members have harmonious relationships, and siblings get along well.

A Strongly agree B agree C Neutral D Agree E strongly disagree

**School Factor**

SF 1. The dormitory environment (hygiene, roommate relationships, etc.) makes me feel comfortable.

A Strongly agree B Agree C Neutral D Agree E Strongly disagree

SF 2. The learning atmosphere in my class is well-constructed.

A Strongly agree B Agree C Neutral D Agree E Strongly disagree

SF 3. I am very satisfied with the natural environment of my school.

A Strongly agree B Agree C Neutral D Agree E Strongly disagree

SF 4. I am very satisfied with the cultural environment (teaching style, learning style, cultural atmosphere, etc.) at my school.

A Strongly agree B Agree C Neutral D Agree E Strongly disagree
SF 5. When I need care from teachers, they respond promptly.

A Strongly agree B Agree C Neutral D Agree E Strongly disagree

SF 6. The care from teachers has a positive impact on me.

A Strongly agree B Agree C Neutral D Agree E Strongly disagree

SF 7. I have a good teacher-student relationship.

A Strongly agree B agree C Neutral D Agree E strongly disagree

SF 8. I can obtain the necessary training and learning opportunities to improve my skills and knowledge.

A Strongly agree B agree C Neutral D Agree E strongly disagree

SF 9. The school provides me with enough opportunities to showcase and utilize my abilities and potential.

A Strongly agree B agree C Neutral D Agree E strongly disagree

SF 10. I can receive fair opportunities and treatment in awards and academic environments.

A Strongly agree B agree C Neutral D Agree E strongly disagree

**Society Factor**

SOF 1. When I encounter difficulties, I can seek help from others online.

A Strongly agree B agree C Neutral D Agree E strongly disagree

SOF 2. I can feel the social support provided by important people around me (e.g., police, doctors, volunteers, etc.).

A Strongly agree B agree C Neutral D Agree E strongly disagree

SOF 3. I have a peer friend with whom I can share my difficulties.

A Strongly agree B agree C Neutral D Agree E strongly disagree

SOF 4. I discuss problem-solving methods with classmates and friends.

A Strongly agree B agree C Neutral D Agree E strongly disagree

SOF 5. I can draw inspiration from unfamiliar peers as role models.

A Strongly agree B agree C Neutral D Agree E strongly disagree

SOF 6. I have relatives with whom I have a very good relationship.

A Strongly agree B agree C Neutral D Agree E strongly disagree

SOF 7. I can feel the understanding, support, and care from one or more relatives.

A Strongly agree B agree C Neutral D Agree E strongly disagree

SOF 8. I feel a strong trust in one or more relatives.

A Strongly agree B agree C Neutral D Agree E strongly disagree
